# Supplementary material for: Spatiotemporally controlled genetic perturbation for efficient large-scale studies of cell non-autonomous effects
Source: eLife. 2018 Nov 27;7:e38393. doi: 10.7554/eLife.38393 (PMC6320068; doi:10.7554/eLife.38393)
Supplement: Transparent reporting form [file elife-38393-transrepform.pdf]

eLIFE
1st Floor
24 Hills Road
Cambridge CB2 1JP, UK
P 01223 855340
W elifesciences.org
T @elife

eLife's transparent reporting form

We encourage authors to provide detailed information *within their submission* to facilitate the interpretation and replication of experiments. Authors can upload supporting documentation to indicate the use of appropriate reporting guidelines for health-related research (see EQUATOR Network), life science research (see the BioSharing Information Resource), or the ARRIVE guidelines for reporting work involving animal research. Where applicable, authors should refer to any relevant reporting standards documents in this form.

If you have any questions, please consult our Journal Policies and/or contact us: editorial@elifesciences.org.

Sample-size estimation

- You should state whether an appropriate sample size was computed when the study was being designed
- You should state the statistical method of sample size computation and any required assumptions
- If no explicit power analysis was used, you should describe how you decided what sample (replicate) size (number) to use

Please outline where this information can be found within the submission (e.g., sections or figure legends), or explain why this information doesn't apply to your submission:

eLife Sciences Publications, Ltd is a limited liability non-profit non-stock corporation incorporated in the State of Delaware, USA, with company number 5030732, and is registered in the UK with company number FC030576 and branch number BR015634 at the address 1st Floor, 24 Hills Road, Cambridge CB2 1JP | August 2014

Previous studies have shown that the number of NSCs per central brain lobe of WT *Drosophila* larva is 90-100 (eg., Lee *et al.* (2006) 439:594-8). Our preliminary data showed a mean of 95 (SD = 3.55, n=13) NSCs per brain lobe. We then used an online sample size calculator (http://www.sample-size.net/sample-size-means/) developed by the UCSF Clinical & Translational Science Institute to calculate the sample size required for our experiments. These were calculated using the T statistic (with a non-centrality parameter.

We input the following requirements:
α (two-tailed) = 0.01 (*Threshold probability for rejecting the null hypothesis. Type I error rate.)*
β = 0.2 (*Probability of failing to reject the null hypothesis under the alternative hypothesis. Type II error rate.)*
q_1 = 0.5 (*Proportion of subjects that are in Group 1 (exposed)*
q_0 = 0.5 (*Proportion of subjects that are in Group 0 (unexposed); 1-q_1)*
E = 10 (*Effect size*)
S = 3.55 (*Standard deviation of the outcome in the population*)

that resulted in the following:
The standard normal deviate for α = Z_α = 2.57583
The standard normal deviate for β = Z_β = 0.84162
Standardized Effect Size = (E/S) = 2.817
Calculation using the T statistic and non-centrality parameter:
N_1: 5, N_0: 5, Total: 10

Note:
The number in effect size was kept low (10) because in a scenario where there is an increased number of NSCs due to tumorigenesis in neural lineages, the number of NSCs are usually too numerous to count. Even an effect size of 30 would give: N_1: 0, N_0: 0, Total: 0 (Sample size). This is because of the small standard deviation of the WT, and the effect size of tumour induction, many standard deviations outside of the mean. Effect size of 30 is 8 standard deviations outside of the mean. Therefore, in this case sample size calculation is unwarranted but we aimed for a minimum of n=5 for each experiment, except for R73G11-FLP; hs-mFLP5, FOFO2.0-pros^{miRs}-GAL4^{miRs} (n=6 from two biological replicates; harder to obtain white prepupa (WPP) stage animals than wandering third-instar larvae (WL3)) in Figure 6. A brief description of the method has been included in the Materials and Methods section.

Sample size is indicated in all figure legends, or within the figure panels in Figure 5 since here we wished to indicate the proportion devoid of EGFP-positive cells in the absence of heat-shock. Sample size is that pooled from independent replicates.

Replicates

- You should report how often each experiment was performed
- You should include a definition of biological versus technical replication

- The data obtained should be provided and sufficient information should be provided to indicate the number of independent biological and/or technical replicates
- If you encountered any outliers, you should describe how these were handled
- Criteria for exclusion/inclusion of data should be clearly stated
- High-throughput sequence data should be uploaded before submission, with a private link for reviewers provided (these are available from both GEO and ArrayExpress)

Please outline where this information can be found within the submission (e.g., sections or figure legends), or explain why this information doesn't apply to your submission:

Each experiment was performed twice (biological replicates). Biological replicates refer to biologically distinct samples (independent crosses) grown in the same conditions and undergone the experimental procedure. Images were acquired using the same confocal (laser power, gain and pinhole) conditions.

Technical replicates would have been recounts of NSC number in the same sample. These were generally not done as the observer experience was that they yielded very similar results.

No data were excluded.

This information is indicated in Materials and Methods under the headings "Immunohistochemistry and imaging" and "Quantifications and statistics".

eLife Sciences Publications, Ltd is a limited liability non-profit non-stock corporation incorporated in the State of Delaware, USA, with company number 5030732, and is registered in the UK with company number FC030576 and branch number BR015634 at the address 1st Floor, 24 Hills Road, Cambridge CB2 1JP | August 2014

Statistical reporting

- Statistical analysis methods should be described and justified
- Raw data should be presented in figures whenever informative to do so (typically when N per group is less than 10)
- For each experiment, you should identify the statistical tests used, exact values of N, definitions of center, methods of multiple test correction, and dispersion and precision measures (e.g., mean, median, SD, SEM, confidence intervals; and, for the major substantive results, a measure of effect size (e.g., Pearson's r, Cohen's d)
- Report exact p-values wherever possible alongside the summary statistics and 95% confidence intervals. These should be reported for all key questions and not only when the p-value is less than 0.05.

Please outline where this information can be found within the submission (e.g., sections or figure legends), or explain why this information doesn't apply to your submission:

Data points shown in Figure 2 – figure supplement1 were collected from two independent biological replicates. The Liliefors test was used to show that the dependent variable (NSC number) of all genotypes was normally distributed. A two-tailed t-test was used to test if any of the means were significantly different from WT. Since this was not the case, one-way analysis of variance (ANOVA) was used to determine whether there were any statistically significant differences between the means of all 7 independent groups (genotypes). Sample size and p-values (derived from Dunnett's multiple comparisons test within ANOVA) are indicated in the legend.

The sample number shown within the panels in Figure 5 were collected from two independent replicates.

Data points shown in Figure 6 were collected from two independent replicates. The Shapiro-Wilk test showed that the 2 dependent variables (normalized tumor volume difference between brain lobes of the same animal and the Sum of tumor volume of both brain lobes in the same animal) of all genotypes except for 1HS Heteroz. were normally distributed. Since not all samples were normally distributed, the Kruskal-Wallis test was used to determine if there were statistically significant differences in the 2 dependent variables between those samples. The number of animals and p-values (derived from Dunn's multiple comparisons test) are indicated in the legend to Figure 6.

Statistical analysis methods can be found in Materials and Methods under the heading "Quantifications and statistics", and the numerical data can be found in Source Data files.

(For large datasets, or papers with a very large number of statistical tests, you may upload a single table file with tests, Ns, etc., with reference to sections in the manuscript.)

Group allocation

- Indicate how samples were allocated into experimental groups (in the case of clinical studies, please specify allocation to treatment method); if randomization was used, please also state if restricted randomization was applied

- Indicate if masking was used during group allocation, data collection and/or data analysis

Please outline where this information can be found within the submission (e.g., sections or figure legends), or explain why this information doesn't apply to your submission:

Samples were allocated into experimental groups according to their genotypes. During data/image acquisition using confocal microscopy, one brain lobe (out of two) for each independent sample was selected at random for Fig 2 – figure supplement 1 while both brain lobes were imaged for volume quantifications shown in Figure 6.
NSC counts and tumor volume quantifications were performed by a blinded observer.

Additional data files ("source data")

- We encourage you to upload relevant additional data files, such as numerical data that are represented as a graph in a figure, or as a summary table
- Where provided, these should be in the most useful format, and they can be uploaded as "Source data" files linked to a main figure or table
- Include model definition files including the full list of parameters used
- Include code used for data analysis (e.g., R, MatLab)
- Avoid stating that data files are "available upon request"

Please indicate the figures or tables for which source data files have been provided:

Figure 2 – figure supplement 1.
Figure 6.
